# A Hybrid Deep Learning Architecture for Content Request Prediction in the Internet of Vehicles

**DOI:** 10.3390/s26103252

**Published:** 2026-05-20

**Authors:** Assem Rezki, Lyamine Guezouli, Abderrezak Benyahia, Djallel Eddine Boubiche, Mohamed Zohir Mabane, Sohaib Chine, Homero Toral-Cruz, Rafael Martínez-Peláez, Julio Cesar Ramirez-Pacheco

**Affiliations:** 1 LaSTIC Laboratory, Computer Science Department, University of Batna 2, Batna 05000, Algeria; a.rezki@univ-batna2.dz (A.R.);; 2 LEREESI Laboratory, HNS-RE2SD, Batna 05000, Algeria; 3 Departamento de Ingeniería y Tecnología, Universidad Autónoma del Estado de Quintana Roo, Chetumal 77019, Mexico; 4 Unidad Académica de Computación, Universidad Politécnica de Sinaloa, Mazatlán 82199, Mexico; 5 Departamento de Ingeniería de Sistemas y Computación, Universidad Católica del Norte, Antofagasta 1270709, Chile; 6 Departamento de Ciencias de la Salud y Tecnología, Universidad Autónoma del Estado de Quintana Roo, Cancún 77519, Mexico

**Keywords:** Internet of Vehicles (IoV), content request prediction, reinforcement learning, caching management

## Abstract

Low-latency content delivery is essential in the Internet of Vehicles (IoV) to support autonomous driving, cooperative perception, and infotainment services. However, rapidly changing vehicular mobility and demand patterns limit the effectiveness of existing content prediction and caching strategies, which often capture either short-term temporal trends or long-range dependencies, but not both. This paper proposes a hybrid deep learning architecture that integrates Long Short-Term Memory (LSTM) networks with Transformer encoders to jointly model fine-grained temporal dynamics and global correlations in content requests. The resulting popularity predictions are incorporated into a reinforcement learning (RL)-based caching policy, enabling proactive and adaptive cache placement at roadside units (RSUs) within an end-to-end optimization framework. Simulation results across representative IoV scenarios show that the proposed approach consistently improves cache hit ratio, retrieval latency, and prediction accuracy compared with LSTM-only, Transformer-only, Least Frequently Used (LFU), and Least Recently Used (LRU) baselines. Ablation studies further demonstrate the complementary strengths of the hybrid components, highlighting improved convergence behavior and robustness under varying demand distributions.

## 1. Introduction

The Internet of Vehicles (IoV) has become a key enabler of intelligent transportation systems (ITSs), supporting seamless communication among vehicles, infrastructure, and pedestrians to enhance road safety, traffic efficiency, and user experience [1]. IoV systems integrate vehicular networks with advanced sensing, wireless communication, and edge computing capabilities. These systems produce and exchange vast amounts of data in highly dynamic and heterogeneous environments [2]. High mobility, rapidly changing topologies, and the real-time nature of vehicular applications introduce significant challenges for timely and reliable data processing [3]. Ensuring low-latency responses is especially critical for safety-critical and context-aware services. Accordingly, machine learning techniques have been increasingly adopted to extract actionable insights from complex data flows and enable predictive analytics, adaptive decision-making, and efficient content dissemination in IoV networks [4]. Comprehensive surveys (e.g., [5]) underscore the growing role of Artificial Intelligence (AI), Machine Learning (ML) and edge-assisted architectures in enabling next-generation connected and autonomous vehicular services under high mobility and stringent latency constraints.

Edge caching is one of the most widely studied mechanisms for reducing latency and alleviating bandwidth pressure in vehicular environments. By storing frequently requested content at RSUs, edge nodes, or on-board storage, IoV systems can reduce reliance on remote servers and improve response times, an important aspect of responsiveness for safety-related and infotainment services [6]. However, traditional caching strategies such as Least Recently Used (LRU), Least Frequently Used (LFU), and static popularity-based schemes struggle to adapt to rapid spatial–temporal fluctuations in content demand. Their reactive nature often leads to suboptimal cache utilization and increased retrieval latency [7]. Likewise, existing content request prediction models tend to capture either short-term variations or long-range global trends, limiting their effectiveness in guiding caching decisions within highly dynamic IoV environments.

Current approaches to IoV caching generally rely on popularity-based strategies or learning-based methods with limited adaptability under rapid mobility and demand fluctuations. Even advanced RL-based schemes (including multi-agent DRL caching frameworks) often rely on demand representations that do not explicitly model both short-term request variations and longer-range dependencies, which can restrict performance in highly dynamic IoV environments [8,9]. Similarly, Priority-Aware Multi-Agent Deep Reinforcement Learning (PA-MADRL) has been proposed for Cellular-Vehicle-to-Anything (C-V2X) resource allocation [10]. Models optimized for short-term prediction may fail to generalize over longer horizons, whereas models focused on global correlations may overlook sudden, localized shifts in demand. These limitations can lead to inaccurate popularity forecasts, suboptimal cache replacement decisions, and increased reliance on remote content retrieval. Moreover, recent IoV studies addressing diffusion or topology optimization primarily target delivery efficiency rather than explicitly modeling temporal demand patterns [11]. In this context, a unified framework that (i) captures short-term dynamics and long-range correlations in request sequences and (ii) couples these predictions into an RL-driven caching control loop remains insufficiently characterized in terms of end-to-end integration details and controlled ablation evidence.

To address these challenges, this paper proposes an integrated framework that combines a hybrid deep learning predictor with an RL-driven caching controller for proactive content placement in IoV networks. The proposed predictor integrates Long Short-Term Memory (LSTM) networks with Transformer architectures to forecast content demand: the LSTM captures short-term temporal dependencies in vehicular request sequences, while the Transformer encoder models long-range correlations and global contextual patterns. Building on these predictions, our framework incorporates the predicted popularity vector into the Deep Q-Network (DQN) state to enable prediction-in-the-loop caching decisions under dynamic IoV conditions. We evaluate the proposed integrated framework end-to-end against classical caching heuristics and predictor baselines. By coupling accurate and adaptive popularity forecasting with proactive caching control, the proposed approach aims to reduce content retrieval latency, increase cache efficiency, and enhance overall network performance in dynamic IoV environments. This study therefore aims to answer the following question: How can hybrid deep learning architectures improve content request prediction and caching efficiency in dynamic IoV environments?

## 2. Related Works

### 2.1. Traditional Caching

Traditional caching strategies, such as Least Recently Used (LRU), Least Frequently Used (LFU), and cooperative caching, have long served as foundational techniques for content delivery in mobile and vehicular networks. These schemes aim to reduce access latency by storing frequently requested content locally, but typically rely on reactive policies and static placement decisions. While these approaches remain relevant, their performance in IoV is limited due to high mobility, dynamic content demand, and volatile network topology.

Recent works have sought to enhance these classical strategies by incorporating contextual awareness. For example, Chen et al. [12] proposed a caching scheme for Vehicular Named Data Networks (VNDNs) that considers the spatial–temporal characteristics of different message types (e.g., safety, traffic, infotainment), leading to improved hit rates and reduced hop counts. Similarly, Gupta et al. [13] introduced a cooperative caching framework that jointly considers content popularity and predicted future ratings using lightweight matrix factorization, still rooted in traditional caching logic. These approaches extend conventional heuristics without employing deep predictive learning.

Nevertheless, traditional and heuristic-based caching strategies continue to face limitations in dynamic vehicular environments. Their reactive nature and reliance on past access patterns make them poorly suited to environments where content popularity shifts rapidly with time and location. As demonstrated by Guezouli et al. [14] and Jin et al. [15], such strategies often underperform under mobility-induced link variability or static placement assumptions. While cooperative caching and simple popularity-based heuristics help improve baseline performance, they lack the adaptability needed for real-time vehicular networks. These challenges motivate the shift toward predictive and learning-driven caching strategies, which are explored in the following subsections.

### 2.2. Learning-Based Prediction

Learning-based caching strategies have emerged to address the limitations of reactive and popularity-based methods by predicting future content demand using machine learning. In IoV environments, where content popularity and mobility are highly dynamic, models such as LSTM, RNN, and transfer learning are increasingly used to support proactive caching.

Several works adopt sequence-based prediction. Wang et al. [16] introduced CCCRP, which uses LSTM to forecast vehicle content requests, followed by reinforcement learning for cache decision-making. Similarly, Zhang et al. [17] applied LSTM for mobility modeling, combining it with a user preference predictor that factors in social proximity and content type. These approaches improve cache freshness and latency, but often rely on centralized data and overlook distributed coordination.

Beyond sequence learning, advanced ML techniques have also been applied. Ashraf et al. [18] used deep transfer learning (DTL) to share content popularity knowledge across vehicular clusters under changing traffic conditions. Zhao et al. [19] proposed a spatiotemporal clustering framework and applied a lightweight multi-armed bandit algorithm for adaptive, real-time caching under connectivity constraints. Li et al. [20] proposed a digital twin-assisted caching system that uses informer-based forecasting to simulate environmental changes and inform proactive cache placement.

While these methods enhance demand forecasting, they typically operate independently of cache control logic and rarely incorporate distributed optimization. This disconnection between prediction and decision-making motivates reinforcement learning-based frameworks, which jointly learn to forecast and act. These are discussed in the next subsection.

### 2.3. Deep Reinforcement Learning and Hybrid Caching Models

To address the limitations of heuristic and prediction-only caching strategies, deep reinforcement learning (DRL) has been widely adopted to enable adaptive cache decision-making in dynamic IoV environments. DRL-based approaches model caching as a sequential decision problem, allowing agents to learn optimal policies through interaction with time-varying vehicular networks.

Early DRL-based caching studies focus on cooperative and multi-agent formulations. Zhang et al. [21] proposed a multi-agent DRL framework in which vehicles act as autonomous agents that jointly optimize content caching and access decisions to minimize distribution delay. Similarly, Zhou et al. [22] developed a distributed deep multi-agent reinforcement learning scheme that explicitly accounts for inter-agent interactions, demonstrating improved cache hit rates over tabular RL and heuristic baselines. Knari et al. [23] further explored cooperative caching using multi-agent DRL, emphasizing resource sharing among vehicles to reduce acquisition latency. These works confirm the effectiveness of DRL in handling dynamic environments, yet generally assume simplified or externally provided content demand models.

More recent studies integrate DRL with advanced system architectures and contextual awareness. UAV-assisted caching combined with federated DRL has been investigated in [24] to jointly optimize caching and computation offloading while preserving user privacy. Chen et al. [25] introduced a social-aware collaborative caching framework that combines dueling double DQN with digital twin networks to cope with complex topology dynamics. Content placement and collaborative caching have also been formulated as DRL problems in [26], where proactive content segmentation improves robustness against intermittent connectivity. Graph-based multi-agent reinforcement learning has been explored in GAMA-Cache [27], where graph attention networks enhance cooperation among edge nodes.

Beyond pure DRL, hybrid frameworks that couple learning-based prediction with DRL-based decision-making have emerged. Jiang et al. [28] combined asynchronous federated learning with DRL to predict content popularity under mobility uncertainty and subsequently optimize caching decisions. Singh et al. [29] proposed a federated DRL framework using a modified PPO algorithm to improve cache efficiency under heterogeneous vehicular densities. Similarly, Wu et al. [30] integrated RNN-based connectivity prediction and multihead attention popularity modeling into a federated multi-agent DRL caching strategy. A secure asynchronous federated learning and DRL hybrid approach was further introduced in [31], leveraging generative models for popularity prediction and multi-agent DRL for proactive caching under privacy constraints.

Despite these advances, most existing DRL-based and hybrid approaches either rely on a single prediction model or loosely couple demand forecasting with caching control. In contrast, the proposed method integrates a hybrid LSTM–Transformer predictor with a reinforcement learning-based caching policy in a unified loop, enabling the joint modeling of short-term temporal dynamics, long-range dependencies, and adaptive cache decision-making.

Taken together, these observations indicate that, despite the substantial progress achieved by deep reinforcement learning-based and hybrid caching frameworks, several limitations remain. Most existing DRL-based approaches assume simplified or externally provided content demand models, while hybrid methods often rely on a single prediction technique and loosely couple demand forecasting with caching control. As a result, these solutions struggle to simultaneously capture short-term temporal fluctuations and long-range dependencies in vehicular content requests, which are critical in highly dynamic IoV environments. Moreover, the interaction between content prediction and caching decision-making is frequently indirect, preventing true end-to-end optimization. These limitations motivate the need for a unified caching framework that tightly integrates expressive content request prediction with adaptive cache control. Accordingly, this work proposes a hybrid LSTM–Transformer prediction model combined with a reinforcement learning-based caching strategy, enabling joint modeling of local temporal dynamics, global attention patterns, and proactive caching decisions in a closed-loop manner.

## 3. The Proposed Framework

### 3.1. Overview and Motivation

The Internet of Vehicles (IoV) operates in highly dynamic environments where vehicular mobility, traffic density, and user interests evolve continuously over time. These characteristics render content demand patterns inherently non-stationary, posing significant challenges to accurate content request prediction and efficient cache management at the network edge. Caching strategies that rely solely on instantaneous observations or historical averages often fail to adapt promptly to rapid variations in content popularity, leading to suboptimal cache utilization and increased access latency.

A fundamental requirement for proactive caching in IoV networks is the ability to forecast future content requests with high accuracy. However, most existing IoV caching frameworks either employ short-term sequence models or long-range attention mechanisms in isolation, and often decouple demand prediction from caching control, which limits their effectiveness in highly dynamic vehicular environments. Sequence-aware models such as Long Short-Term Memory (LSTM) networks are effective in capturing local temporal variations in content requests, but their ability to model long-range dependencies is limited. Conversely, attention-based architectures such as Transformers excel at learning global correlations over long sequences, yet may overlook fine-grained temporal continuity when applied directly to raw request data.

**Figure 1:**
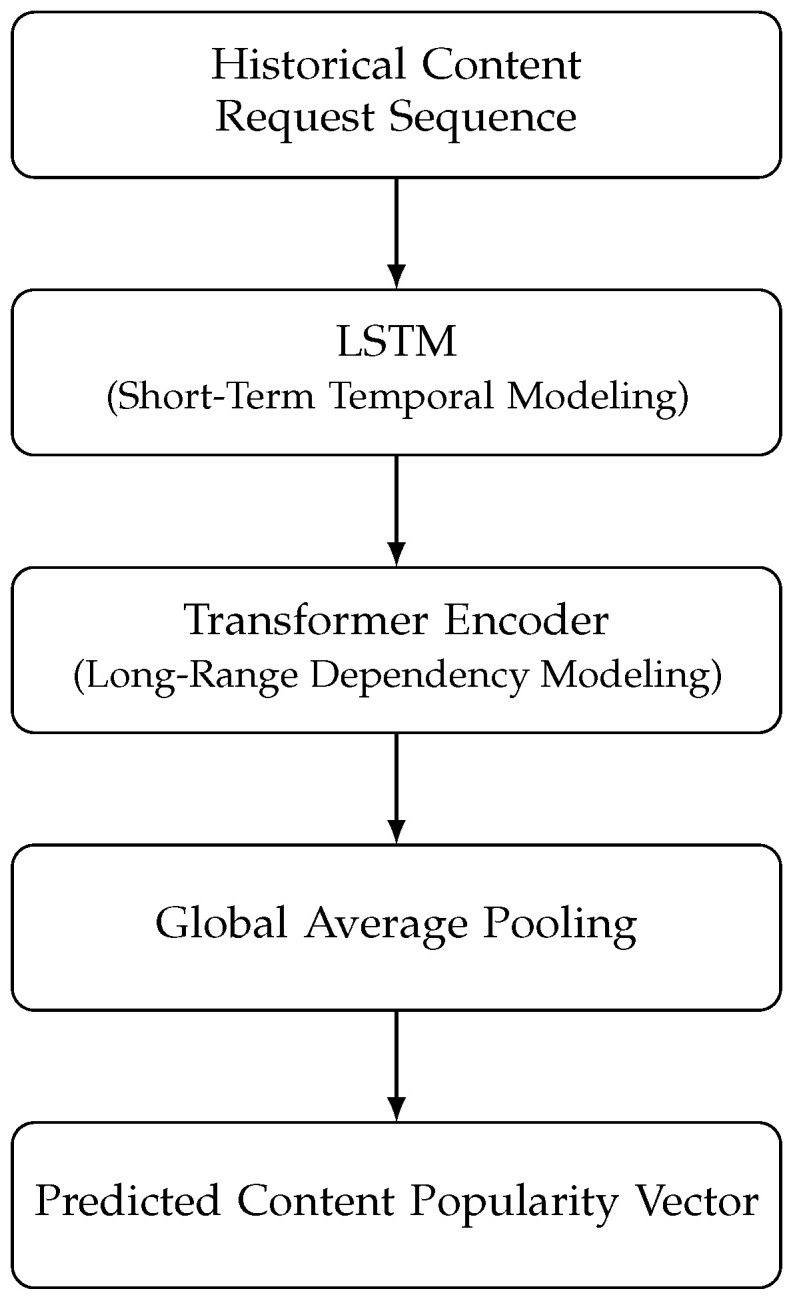
Hybrid LSTM–Transformer architecture for content request prediction in the Internet of Vehicles (IoV). The LSTM captures short-term temporal dynamics from historical request sequences, while the Transformer encoder models long-range dependencies via self-attention. Global average pooling aggregates temporal features into a compact popularity representation.

To address these complementary limitations, this work introduces a hybrid deep learning architecture that integrates LSTM networks with Transformer encoders for content request prediction. As illustrated in Figure 1, the proposed hybrid predictor first applies an LSTM to encode short-term request dynamics, followed by a Transformer encoder that captures long-range dependencies across the observation window. This serial design allows the model to jointly exploit local temporal continuity and global attention-based relationships in vehicular content demand. Placing the LSTM before the Transformer allows short-term temporal noise and local fluctuations to be smoothed into structured latent representations, which subsequently serve as more informative and stable tokens for the attention mechanism. This ordering is adopted as an inductive-bias design choice for nonstationary IoV request sequences; while we provide component-wise comparisons against LSTM-only and Transformer-only predictors in Section 4, an explicit architecture-order ablation (e.g., Transformer-first or parallel fusion) is identified as an important direction for extended evaluation.

While accurate prediction is a necessary enabler, prediction alone is insufficient to guarantee efficient caching unless it is tightly coupled with cache decision-making. Caching actions directly influence future content availability and observed request patterns, which in turn affect subsequent predictions. Motivated by this observation, the proposed framework embeds the hybrid LSTM–Transformer predictor within a reinforcement learning-based caching control process. Specifically, predicted content popularity is incorporated into the decision state of a deep reinforcement learning agent, enabling proactive and adaptive cache placement and replacement decisions.

**Figure 2:**
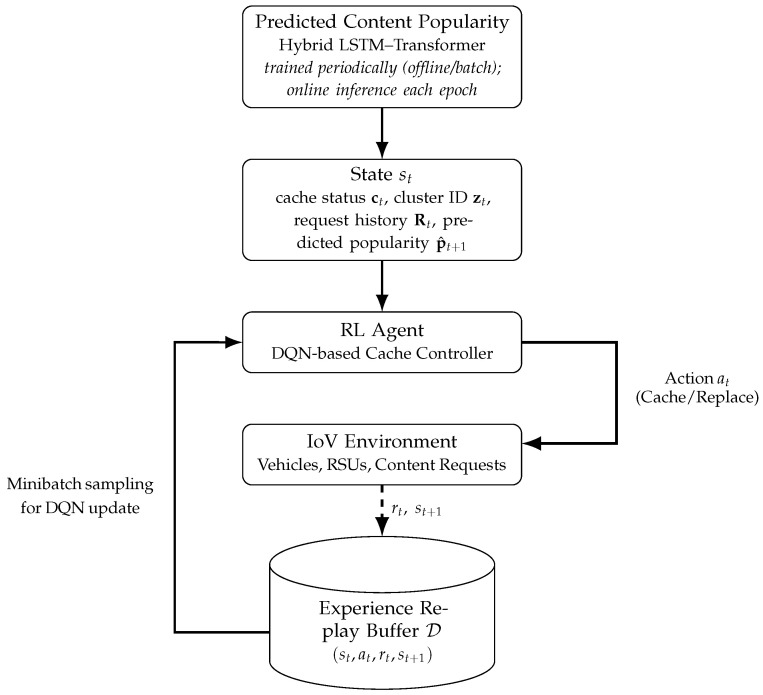
Closed-loop interaction between hybrid LSTM–Transformer-based content popularity prediction and DQN-based cache management in IoV. Predicted popularity is incorporated into the RL state, while caching decisions affect future system states and rewards through the vehicular environment. Here, “offline/batch” refers to periodic training updates of the predictor, whereas the predictor output is computed by online inference at each decision epoch and injected into the DQN state.

This interaction establishes a closed-loop learning framework, depicted in Figure 2, in which content popularity prediction and caching control continuously inform each other through system feedback. The remainder of Section 3 elaborates on the individual components of this framework, beginning with the hybrid LSTM–Transformer model for content request prediction, followed by the reinforcement learning formulation for cache management and their closed-loop integration.

### 3.2. Hybrid LSTM–Transformer Model for Content Request Prediction

This subsection presents the hybrid prediction model illustrated in Figure 1, which is designed to forecast future content demand in Internet of Vehicles (IoV) environments based on historical request observations. Let $X_{t-L+1:t}=\left\{x_{t-L+1},x_{t-L+2},\ldots ,x_{t}\right\}$ denote the sequence of observed content request features within an observation window of length *L*. Each vector $x_{\tau }\in R^{K}$ represents a feature-based description of content requests at time step τ, where *K* denotes the number of contents considered in the system. Both the observation window length *L* and the catalog size *K* are treated as configurable parameters rather than fixed constants, and their specific values depend on the simulation scenario and dataset used in the experimental evaluation, as detailed in Section 4. The representation of $x_{\tau }$ may depend on the application scenario and can encode request counts, normalized popularity indicators, or other aggregated request-related features.

The goal of the prediction model is to estimate the relative popularity of contents at the next decision epoch, expressed as a predicted popularity vector ${\hat{p}}_{t+1}\in R^{K}$. Each element ${\hat{p}}_{t+1}^{\left(i\right)}$ reflects the predicted demand tendency of content *i* rather than an exact request value. This vector provides a compact and forward-looking summary of expected content demand and is later incorporated into the cache management process.

As depicted in Figure 1, the proposed predictor adopts a serial hybrid architecture. First, a Long Short-Term Memory (LSTM) network processes the input sequence $X_{t-L+1:t}$ to capture short-term temporal dependencies and local request dynamics. The resulting hidden representations are then passed to a Transformer encoder, which applies self-attention mechanisms to model long-range dependencies and global correlations across the observation window. To obtain a fixed-dimensional representation suitable for downstream use, a global average pooling operation is applied over the Transformer outputs. The resulting embedding is subsequently mapped to the predicted popularity vector ${\hat{p}}_{t+1}$ through a fully connected layer.

Placing the LSTM before the Transformer serves as an effective temporal feature encoding stage that transforms raw request sequences into smoother latent representations. This preprocessing reduces short-term noise and burstiness in vehicular request data, thereby improving the effectiveness of the subsequent self-attention mechanism in capturing long-range dependencies. Such a serial ordering is particularly suitable for IoV environments, where localized demand fluctuations coexist with broader temporal trends. In this work, the serial LSTM-first, Transformer-later structure is therefore motivated by stability and inductive bias rather than claimed as uniquely optimal. A systematic comparison with alternative hybrid configurations under matched parameter and training budgets remains part of extended evaluation.

#### 3.2.1. LSTM-Based Short-Term Temporal Encoding

The first stage of the hybrid predictor focuses on modeling short-term temporal dynamics in content request sequences. Given the input sequence $X_{t-L+1:t}=\left\{x_{t-L+1},\ldots ,x_{t}\right\}$, the Long Short-Term Memory (LSTM) network processes the observations sequentially to capture local temporal dependencies and continuity in request patterns.

At each time step τ∈{t−L+1,…,t}, the LSTM updates its internal state according to

(1) $$h_{\tau },c_{\tau }=\mathrm{LSTM}\left(x_{\tau },h_{\tau -1},c_{\tau -1}\right),$$

where $h_{\tau }\in R^{d_{h}}$ denotes the hidden state vector, $c_{\tau }\in R^{d_{h}}$ is the memory cell state, and $d_{h}$ is the dimensionality of the LSTM hidden representation. Through its gated structure, the LSTM selectively retains or forgets historical information, enabling it to effectively model short-term request fluctuations caused by transient vehicular behaviors such as traffic congestion or localized user interest.

Rather than directly using the final hidden state for prediction, the sequence of LSTM hidden states $\left\{h_{t-L+1},\ldots ,h_{t}\right\}$ is preserved and forwarded to the subsequent Transformer encoder. This design choice allows short-term temporal features extracted by the LSTM to serve as enriched token representations for the attention mechanism, thereby facilitating joint modeling of local temporal continuity and global request correlations in later stages.

#### 3.2.2. Transformer Encoder for Long-Range Dependency Modeling

Although the LSTM module effectively captures short-term temporal continuity in content request sequences, it is inherently limited in modeling long-range dependencies when the observation window becomes large. In highly dynamic IoV environments, content popularity may be influenced by non-adjacent historical events, recurring traffic patterns, or delayed user interests that cannot be adequately captured by local temporal modeling alone. To address this limitation, the proposed hybrid architecture incorporates a Transformer encoder following the LSTM stage.

Let $H=\{h_{t-L+1},h_{t-L+2},\ldots ,h_{t}\}$ denote the sequence of hidden representations produced by the LSTM. These representations, which already encode short-term temporal dynamics, are treated as input tokens to the Transformer encoder. By applying self-attention over this sequence, the Transformer explicitly models global correlations across the entire observation window, allowing the predictor to identify long-range temporal dependencies among content request patterns.

The self-attention mechanism is formally expressed as

(2) $$\mathrm{Attention}\left(Q,K,V\right)=\mathrm{softmax}\;\left(\frac{QK^{⊤}}{\sqrt{d_{k}}}\right)V,$$

where Q, K, and V denote the query, key, and value matrices obtained through linear projections of H, and $d_{k}$ is the dimensionality of the key vectors. This operation enables each time step to selectively attend to all other time steps in the sequence, thereby capturing long-range dependency structures that may span across distant temporal positions.

The output of the Transformer encoder is a refined sequence of representations $\tilde{H}=\left\{{\tilde{h}}_{t-L+1},\ldots ,{\tilde{h}}_{t}\right\}$, which jointly encodes local temporal continuity inherited from the LSTM and global dependency information captured through self-attention. Architectural hyperparameters, including the number of encoder layers, attention heads, and hidden dimensions, are omitted here for clarity and specified in Section 4. For fair comparison, the Transformer-only baseline uses the same Transformer configuration as the hybrid model.

#### 3.2.3. Temporal Feature Aggregation and Popularity Vector Prediction

The Transformer encoder produces a refined sequence of contextualized representations $\tilde{H}=\left\{{\tilde{h}}_{t-L+1},{\tilde{h}}_{t-L+2},\ldots ,{\tilde{h}}_{t}\right\}$, where each ${\tilde{h}}_{\tau }\in R^{d_{h}}$ encodes both short-term temporal dynamics and long-range dependency information. However, reinforcement learning-based cache control requires a fixed-dimensional representation of future demand rather than a variable-length sequence. To this end, a temporal aggregation mechanism is applied to summarize the sequence into a compact embedding.

Specifically, global average pooling is employed to aggregate the Transformer outputs across the temporal dimension, yielding

(3) $$z_{t}=\frac{1}{L}\sum_{\tau =t-L+1}^{t}{\tilde{h}}_{\tau },$$

where $z_{t}\in R^{d_{h}}$ represents a compact embedding that summarizes the overall evolution of content request patterns within the observation window. Compared with attention-based or position-specific pooling, global average pooling provides a simple and stable aggregation strategy while avoiding additional trainable parameters.

The aggregated embedding $z_{t}$ is then mapped to a predicted content popularity vector through a fully connected output layer, expressed as

(4) $${\hat{p}}_{t+1}=f_{\mathrm{out}}\left(z_{t}\right)=W_{o}z_{t}+b_{o},$$

where $W_{o}\in R^{K\times d_{h}}$ and $b_{o}\in R^{K}$ are trainable parameters, and *K* denotes the number of contents in the catalog. The resulting vector ${\hat{p}}_{t+1}\in R^{K}$ represents the predicted relative popularity of contents at the next decision epoch.

This predicted popularity vector serves as the final output of the hybrid LSTM–Transformer predictor and provides a forward-looking representation of content demand. In the subsequent subsection, this prediction is incorporated into the state definition of the reinforcement learning agent to enable proactive and adaptive cache management decisions.

### 3.3. Reinforcement Learning-Based Cache Management

#### 3.3.1. MDP Formulation and State Definition

To translate predicted content demand into proactive caching decisions under dynamic IoV conditions, the cache management problem is formulated as a Markov decision process (MDP) controlled by a DQN agent. As illustrated in Figure 2, at each decision epoch *t*, the agent observes the system state $s_{t}$, selects a caching action $a_{t}$, and receives a scalar reward $r_{t}$ that reflects the effectiveness of the decision in terms of content delivery performance and resource utilization.

Figure 2 indicates that the hybrid predictor is trained periodically using offline/batch training. We clarify that this refers to the parameter updates of the LSTM–Transformer predictor, which are performed intermittently (e.g., after a fixed number of episodes or once sufficient new request data is accumulated). In contrast, during simulation runtime the predictor is used in online inference mode at every decision epoch *t* to compute ${\hat{p}}_{t+1}$ from the most recent request-history window. The DQN controller then uses this predicted demand information within $s_{t}$ to select caching actions, while the DQN itself is updated online via experience replay and target-network synchronization. Therefore, the framework is “closed-loop” at the control level (prediction-in-the-loop for decision making), although predictor training can be performed periodically in batch.

The state $s_{t}$ is designed to capture both instantaneous system conditions and forward-looking demand information. Specifically, it aggregates three components: (i) the current cache status at the controlled node (e.g., RSU or vehicle), (ii) lightweight network or context indicators, and (iii) the predicted content popularity vector ${\hat{p}}_{t+1}$ generated by the hybrid LSTM–Transformer model described in Section 3.2.3 A representative state vector is expressed as

(5) $$s_{t}=\left[\;c_{t}\;;\;n_{t}\;;\;{\hat{p}}_{t+1}\;\right],$$

where $c_{t}$ encodes the cache occupancy (e.g., binary indicators or normalized cache fractions per content), and $n_{t}$ summarizes local network context information available to the node (such as traffic load, mobility-related indicators, or contact-duration proxies). In addition, $z_{t}\in {\left\{0,1\right\}}^{C}$ represents the one-hot encoded cluster membership of the controlled vehicle or RSU, capturing its spatial or logical grouping within the IoV environment. Here, $z_{t}$ is included as an auxiliary mobility/context feature (derived from speed-level clustering) that can correlate with contact opportunities and dissemination stability, rather than as a definitive descriptor of similarity in vehicles’ content request preferences. The primary forward-looking demand signal in the state remains the predicted popularity vector ${\hat{p}}_{t+1}$.

**Figure 3:**
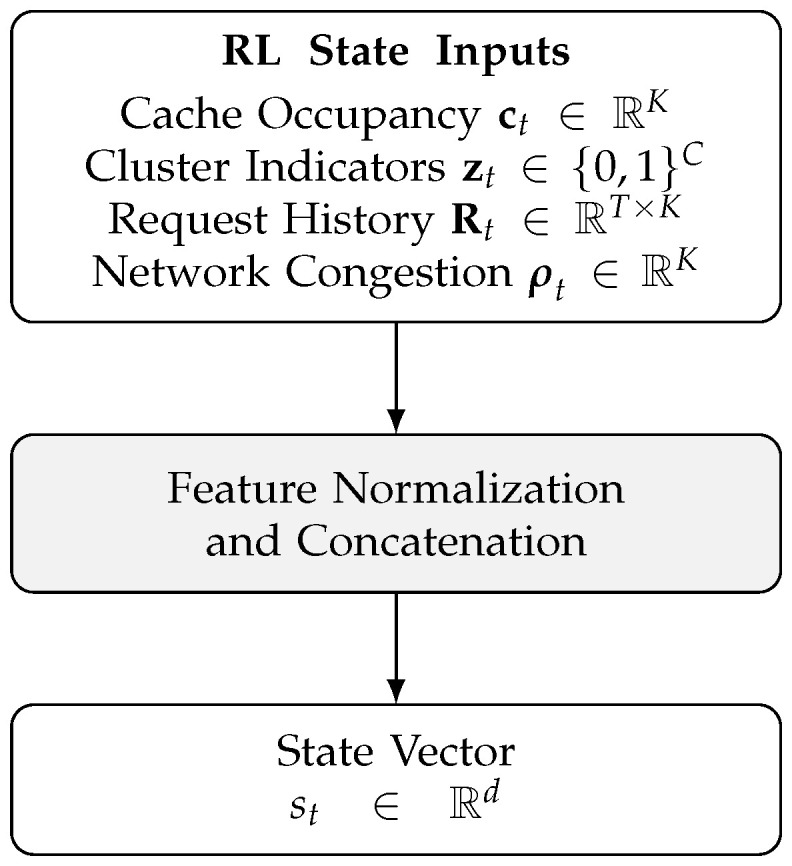
Construction of the RL state vector. Heterogeneous features describing cache occupancy, cluster membership, historical content requests, and network congestion are normalized and concatenated to form the Markov state $s_{t}$ used by the DQN agent.

As illustrated in Figure 3 and formally summarized in Algorithm 1, all state components, including $c_{t}$, $z_{t}$, request history features, and network indicators, are normalized and concatenated to construct the final RL state vector $s_{t}$.
**Algorithm 1** Construction of the Reinforcement Learning State Vector**Require:** $C\in {\left\{0,1\right\}}^{M\times K}$▷ Cache occupancy matrix,  1:  $z\in {\left\{0,1\right\}}^{C}$▷ One-hot mobility-context cluster (speed-level based),  2:  $R\in R^{T\times K}$▷ Request history matrix,  3:  $S_{\max}$▷ Maximum cache slots,  4:  $\rho \in R^{K}$▷ Optional congestion metrics**Ensure:** $s\in R^{d}$▷ State vector (d=2K+C+TK)  5:  Compute cache occupancy:  6:      $c\leftarrow \left[\sum_{j=1}^{M}C_{j1}/S_{\max},\ldots ,\sum_{j=1}^{M}C_{jK}/S_{\max}\right]$  7:  Encode cluster indicators:  8:      $z^{\prime }\leftarrow z$▷ One-hot vector (no normalization)  9:  Flatten request history:10:      h←vec(R)11:  Normalize congestion (if provided):12:  **if** 
ρ≠∅ 
**then**13:         $\rho^{\prime }\leftarrow \rho /\max\left(\rho \right)$14:  **else**15:         $\rho^{\prime }\leftarrow 0_{K}$16:  **end if**17:  Concatenate components:18:      $s\leftarrow [c∥z^{\prime }∥h∥\rho^{\prime }]$19:      $s\leftarrow \left\lfloor \frac{s}{{∥s∥}_{1}}\right\rfloor$20:  **return** *s*

Formally, the cache management problem is modeled as a Markov decision process (MDP) 〈S,A,P,R,γ〉, where S denotes the state space defined above, A is the action space described in the following subsection, R corresponds to the reward function defined later, P captures the environment transition dynamics, and γ∈(0,1) is the discount factor.

Algorithm 1 computes cache occupancy by summing over the cache-occupancy matrix $C\in {\left\{0,1\right\}}^{M\times K}$, which costs O(MK). Flattening the request-history matrix $R\in R^{T\times K}$ costs O(TK), and normalizing the congestion vector $\rho \in R^{K}$ costs O(K). Concatenation and $\ell_{1}$ normalization operate over d=2K+C+TK features, costing O(d). Therefore, the overall upper-bound time complexity per state construction is

$$O\left(MK+TK+C+K\right)=O\;\left(K(M+T)+C\right).$$

Depending on the deployment scenario, the state can be defined at the vehicle, RSU, or local cluster level without changing the learning formulation. This design explicitly realizes the closed-loop interaction between prediction and caching: the popularity predictor provides anticipatory information on future demand, while the cache status and network context reflect current system constraints that influence feasible caching actions. To ensure numerical stability and consistent scaling across heterogeneous state components, normalization is applied during RL state construction before feeding $s_{t}$ into the Q-network.

#### 3.3.2. Action Definition

At each decision epoch *t*, the DQN agent selects a caching action $a_{t}$ based on the observed state $s_{t}$. The action determines how the cache content at the controlled node (e.g., RSU or vehicle) is updated in response to predicted content demand and current system constraints.

In this work, the action space is defined to support cache placement and replacement decisions under limited storage capacity. Specifically, an action $a_{t}$ corresponds to selecting a candidate content item to be cached, potentially triggering the eviction of an existing item when the cache is full. The selection is guided by the predicted popularity vector ${\hat{p}}_{t+1}$ and the current cache state $c_{t}$, allowing the agent to proactively prioritize contents expected to be requested in the near future.

To ensure compatibility with DQN-based learning and avoid excessive complexity, the action space is discrete and constrained by cache feasibility rules (e.g., capacity limits and duplicate avoidance). The overall action selection and learning procedure, including exploration, reward feedback, and Q-network updates, is detailed in Algorithm 2, which governs the end-to-end caching optimization process. The action space cardinality scales linearly with the content catalog size and cache capacity, allowing the formulation to remain tractable while avoiding combinatorial explosion in large-scale deployments.
**Algorithm 2** Deep Q-Learning-Based Caching Optimization in Internet of Vehicles.**Require:** $\epsilon_{\mathrm{start}}=1.0$, $\epsilon_{\min}=0.01$, κ=0.995  1:  Replay buffer D capacity N=10,000  2:  Target update frequency τ=100 steps  3:  Batch size B=64  4:  Initialize Q-network $Q_{\theta }$ and target network $Q_{\theta^{-}}$  5:  **for** episode i=1 to *M* **do**  6:     Observe initial state $s_{0}$  7:     **for** t=0 to $T_{\max}$ **do**  8:        With probability $\epsilon_{t}$, choose random feasible action $a_{t}$  9:        Else choose $a_{t}=\arg\max_{a}Q\left(s_{t},a;\theta \right)$10:        Execute $a_{t}$, observe reward $r_{t}$ and next state $s_{t+1}$11:        Store transition $\left(s_{t},a_{t},r_{t},s_{t+1}\right)$ in D12:        Sample minibatch of *B* transitions from D13:        **for** each sampled transition $\left(s_{j},a_{j},r_{j},s_{j}^{\prime }\right)$ **do**14:              **if** $s_{j}^{\prime }$ is terminal **then**15:              $y_{j}\leftarrow r_{j}$16:              **else**17:              $y_{j}\leftarrow r_{j}+\gamma \max_{a^{\prime }}Q\left(s_{j}^{\prime },a^{\prime };\theta^{-}\right)$18:              **end if**19:        **end for**20:        Update θ to minimize $\frac{1}{B}\sum_{j}{\left(y_{j}-Q\left(s_{j},a_{j};\theta \right)\right)}^{2}$21:        Update exploration: $\epsilon_{t+1}=\max(\epsilon_{\min},\epsilon_{t}\cdot \kappa )$22:        **if** tmodτ=0 **then**23:              Update target network: $\theta^{-}\leftarrow \theta$24:        **end if**25:     **end for**26:  **end for**


#### 3.3.3. Reward Design

The reward function is designed to guide the learning agent toward caching decisions that improve content delivery efficiency while respecting resource constraints in dynamic IoV environments. At each decision epoch *t*, after executing the selected action $a_{t}$, the agent receives a scalar reward $r_{t}$ reflecting the immediate impact of this decision on system performance.

The reward incorporates three complementary objectives: (i) promoting cache hits, (ii) reducing content acquisition latency, and (iii) encouraging efficient utilization of network and backhaul resources. Accordingly, the reward at time *t* is defined as

(6) $$r_{t}=\alpha \;H_{t}+\beta \;{\tilde{D}}_{t}+\eta \;U_{t},$$

where $H_{t}$ denotes a popularity-weighted cache-hit score computed after executing action $a_{t}$. Let $C_{jk}\left(t\right)\in \left\{0,1\right\}$ indicate whether content item k∈{1,…,K} is stored at caching node j∈{1,…,M} at epoch *t*, where *M* is the number of caching nodes considered in the cooperative cache tier (in our setup, M=3 including the RSU and the cluster vehicles). We define the availability indicator

(7) $$I_{k}^{\mathrm{cached}}\left(t\right)=I\left(\sum_{j=1}^{M}C_{jk}\left(t\right)>0\right),$$

which equals 1 if content *k* is cached in at least one node in the cooperative tier and 0 otherwise. The cache-hit component is then computed as

(8) $$H_{t}=\sum_{k=1}^{K}{\hat{p}}_{t+1,k}\;I_{k}^{\mathrm{cached}}\left(t\right),$$

where ${\hat{p}}_{t+1,k}$ is the predicted relative popularity score of content *k* for the next decision epoch. This formulation provides a dense and stable learning signal that encourages caching contents with high predicted demand.

${\tilde{D}}_{t}$ denotes a normalized latency-reduction term (e.g., ${\tilde{D}}_{t}={\left(1+d_{t}\right)}^{-1}$ with retrieval delay $d_{t}$), and $U_{t}$ captures normalized resource-related benefits such as bandwidth/backhaul savings. The weighting coefficients α, β, and η control the relative importance of these objectives and are selected empirically. Here, $U_{t}$ is used to avoid notation conflicts with the replay buffer and batch size used in Algorithm 2.

All reward components are normalized to [0,1] and combined via a weighted sum with α+β+η=1. Unless otherwise stated, we use a balanced setting (α,β,η)=(0.3,0.4,0.3). To make the reward design reproducible and to support sensitivity analyses in extended evaluations, we also considered a hit-focused setting (0.6,0.2,0.2) and a latency-sensitive setting (0.2,0.6,0.2).

**Figure 4:**
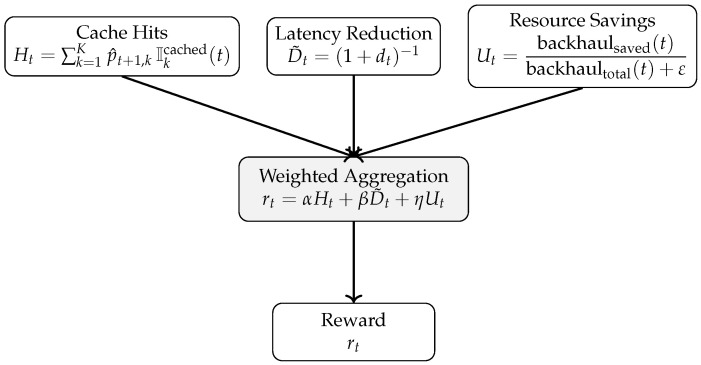
Reward computation in the proposed DQN-based caching framework. The scalar reward is obtained by aggregating cache hits, latency reduction, and resource savings.

As illustrated in Figure 4, the reward signal is computed as a weighted aggregation of cache hits, latency reduction, and resource savings. This decomposition makes the contribution of each performance objective explicit and enables the learning agent to jointly optimize content delivery efficiency and resource utilization while maintaining a simple scalar reward formulation suitable for DQN-based learning.

By relying on observable system feedback rather than idealized demand assumptions, the proposed reward design provides a stable and interpretable learning signal, allowing the agent to adapt its caching policy to varying request patterns and network conditions.

Algorithm 2 runs for *M* episodes and $T_{\max}$ decision epochs per episode. At each epoch, it (i) selects an action by evaluating the Q-network (one forward pass), (ii) samples a minibatch of size *B*, and (iii) performs one gradient update of the Q-network (backpropagation) using that minibatch. Let |θ| denote the number of trainable parameters in the Q-network. A forward/backward pass is O(|θ|) up to constant factors, while computing targets over the minibatch adds an O(B) term (dominated by network evaluation). Thus, the overall upper-bound training complexity is

$$O\;\left(M\;T_{\max}\;B\;\left|\theta \right|\right),$$

and the per-epoch complexity is O(B|θ|).

#### 3.3.4. Training and Update Procedure

The caching policy is learned using a DQN, which approximates the action-value function $Q(s_{t},a_{t})$ over the defined state and action spaces. During training, the agent interacts with the IoV environment in discrete decision epochs, following the closed-loop interaction illustrated in Figure 2. At each time step *t*, the agent observes the current state $s_{t}$, selects a caching action $a_{t}$ according to an ϵ-greedy exploration strategy, executes the corresponding cache placement or replacement decision, and receives the immediate reward $r_{t}$ together with the subsequent state $s_{t+1}$.

Each interaction generates a transition tuple $\left(s_{t},a_{t},r_{t},s_{t+1}\right)$, which is stored in an experience replay buffer. Mini-batches of transitions are uniformly sampled from this buffer during training to break temporal correlations between successive samples and to improve the stability of gradient-based updates. The DQN parameters are optimized by minimizing the temporal-difference (TD) loss between the predicted Q-values and the target Q-values computed using a separate target network, as formalized in Algorithm 2.

To further enhance learning stability, the parameters of the target network are periodically synchronized with those of the online Q-network. This delayed update mechanism mitigates oscillations and divergence during training, which are common issues in value-based reinforcement learning with function approximation. Meanwhile, the exploration rate ϵ is gradually decayed over time, enabling a smooth transition from exploratory behavior to exploitation of the learned caching policy.

Convergence of the learning process is monitored using standard reinforcement learning diagnostics, including the evolution of the TD loss and the moving average of episode returns. These indicators provide empirical evidence of training stability and policy improvement over time, ensuring that the learned caching strategy reliably adapts to dynamic content demand and vehicular mobility conditions. The complete training and update procedure is summarized in Algorithm 2.

In summary, Section 3 has presented an end-to-end IoV caching framework that tightly integrates hybrid content popularity prediction with reinforcement learning-based cache management. The proposed LSTM–Transformer model provides forward-looking demand estimates, which are explicitly incorporated into the state of a DQN agent to enable proactive and adaptive caching decisions under dynamic vehicular conditions. In the following section, we evaluate the effectiveness of the proposed framework through extensive simulations, comparing its performance against representative baseline strategies in terms of cache hit ratio, content acquisition delay, and learning behavior under varying network and demand scenarios.

## 4. Experimental Setup and Results

### 4.1. Simulation Environment

To evaluate the performance of the proposed hybrid model for content request prediction in the Internet of Vehicles (IoV), a custom simulation environment was developed to emulate an urban vehicular scenario under controlled conditions. The objective of this environment is to analyze the interaction between content request prediction and caching decisions, rather than to reproduce a full-scale city-wide deployment. This controlled design enables clear observation of learning dynamics and relative performance trends among competing caching strategies.

Although our simulator is configured as a compact proof-of-concept environment, its purpose is to enable controlled analysis of the interaction between demand prediction and caching decisions under identical conditions. The specific scale parameters and the scope of interpretation of the reported metrics are detailed in Section 4.2.1.

The network scenario is modeled as a single-direction urban road segment covered by multiple Roadside Units (RSUs), with non-overlapping coverage areas. Vehicles travel along the road at discrete speed levels to reflect heterogeneous mobility conditions commonly observed in urban traffic. Specifically, vehicle speeds are divided into $L_{v}$ discrete levels, and each vehicle randomly selects a speed level upon entering the road, moving within the corresponding speed range throughout its trajectory. This discretized mobility model facilitates stable clustering and reduces frequent topology changes caused by continuous speed variations.

To mitigate the impact of high mobility on content transmission stability, vehicles are grouped into clusters based on their speed levels. Each cluster operates within the coverage area of a single RSU, ensuring that no cluster spans multiple RSUs. This design choice allows localized content dissemination and cooperative caching while avoiding inter-RSU handover effects during a single episode. Clustering is performed using the K-means algorithm, which groups vehicles with similar mobility characteristics to enhance cache effectiveness. We note that the purpose of speed-level clustering is to provide a lightweight mobility-context abstraction (e.g., contact stability and interaction locality) rather than to claim perfect similarity in content request preferences. In our simulator, clustering is computed at the beginning of each episode based on the assigned discrete speed levels and is kept fixed within that episode to maintain a controlled and reproducible learning environment.

Each cluster includes caching nodes composed of Cluster Vehicles (CVs) and the associated RSU, enabling both vehicle-assisted and infrastructure-supported content delivery. RSUs act as stable edge caching entities, while CVs provide opportunistic storage within clusters. This hybrid caching topology reflects a typical edge-assisted IoV architecture and supports the evaluation of collaborative caching strategies.

A content repository consisting of multiple content items with heterogeneous sizes is considered to emulate diverse data demands, including infotainment and traffic-related content. Vehicle content requests follow a stochastic arrival process modeled using a Poisson distribution, which captures the bursty and time-varying nature of user requests in vehicular environments. In addition, the Poisson model provides a lightweight and controllable mechanism to vary the request intensity across decision epochs while keeping the arrival process statistically well-defined. This modeling choice is intended to generate temporally dispersed request patterns rather than to reproduce long-tail content popularity at scale.

Request generation combines a Poisson arrival process (to determine how many request arrivals occur per decision epoch) with a Zipf-like content-selection distribution (to sample which content item is requested at each arrival). In our implementation, the Zipf-like sampling probabilities are constructed from the predictor’s ranked outputs (higher-ranked items receive higher selection probability). The resulting request sequence may be generated once at episode initialization or generated sequentially across decision epochs; in both cases, it follows the same Poisson–Zipf procedure.

The simulation is organized into training and testing phases to evaluate both learning behavior and generalization. Detailed episode counts, split ratios, and the full evaluation protocol are provided in Section 4.2.1.

Because speed-level clustering is only a proxy for mobility similarity, it may not fully reflect similarities in vehicles’ content request patterns under highly dynamic IoV conditions; the clustering feature can be extended toward adaptive online clustering and/or request-aware clustering (e.g., using recent request embeddings or learned mobility-demand representations) in future evaluations.

This structured simulation environment ensures reproducibility and controlled analysis of the proposed hybrid architecture. The next subsection details the specific system, network, and learning parameters used throughout the simulations.

### 4.2. Simulation Parameters and Configuration

#### 4.2.1. System Parameters

The simulation environment models a compact and controlled vehicular scenario consisting of 15 vehicles. This scale is intentionally selected to enable stable training of learning-based components and to isolate the effects of content request prediction and caching decisions under dynamic mobility conditions. Similar small-scale configurations are commonly adopted in IoV caching studies as an initial validation step prior to large-scale experimentation.

Vehicles are grouped into 2 clusters using the K-means algorithm based on mobility characteristics, enabling localized caching strategies and reducing the impact of rapid topology changes. Each cluster includes one designated Cluster Vehicle (CV), and the entire system is supported by a fixed Roadside Unit (RSU), resulting in a total of 3 caching nodes. This configuration allows the evaluation of cooperative caching between mobile and infrastructure-based nodes while avoiding excessive system complexity.

Cache capacities are deliberately constrained to 5 MB for vehicles and 10 MB for the RSU to reflect practical memory limitations at edge devices and to challenge the effectiveness of caching policies under resource scarcity. By limiting cache size, the simulation emphasizes intelligent content selection rather than brute-force storage.

The content pool consists of 10 unique content items with heterogeneous sizes, representing a simplified abstraction of infotainment and traffic-related data. While the number of contents is limited, this configuration is sufficient to analyze relative caching performance and decision behavior under constrained conditions. The small content set should therefore be interpreted as a proof-of-concept setting rather than a large-scale content catalog.

Overall, the above configuration (15 vehicles, 10 contents, 1 RSU, and 2 clusters) is intentionally selected as a controlled proof-of-concept to isolate the interaction between content request prediction and caching control and to ensure stable training of learning-based components. Accordingly, the absolute values of hit ratio, delay, and accuracy reported in Section 4 should be interpreted as comparative indicators under identical conditions rather than as performance guarantees for large-scale, high-density IoV deployments.

Content request popularity is generated using a Zipf-like distribution with parameter α=0.7 to emulate skewed access patterns in which a subset of contents dominates user demand. A Zipf-like popularity model is used to emulate skewed access patterns in which a small subset of contents attracts most requests; here it serves as a controlled proxy for popularity imbalance rather than a claim about any specific real-world catalog. Although Zipf distributions are typically associated with large-scale datasets, its use here is limited to modeling relative popularity imbalance rather than long-tail behavior. Consistent with Section 4.1, the Poisson process determines the number of request arrivals per decision epoch, while the content item requested at each arrival is sampled using a Zipf-like probability mass function constructed from the predictor’s ranked outputs (i.e., higher-ranked items are assigned higher selection probability). This yields a Poisson–Zipf request generation mechanism.

A total of 1000 simulation episodes are executed, with the first 500 episodes used for training learning-based models and the remaining 500 episodes reserved for testing. Each episode corresponds to a complete interaction cycle under a fixed mobility realization. This equal split ensures sufficient training convergence while allowing unbiased evaluation of generalization performance. Alternative ratios (e.g., 70/30) were also considered and yielded similar qualitative trends (see Table 1).

#### 4.2.2. Reinforcement Learning

The reinforcement learning (RL) component plays a central role in optimizing content caching decisions in the proposed framework. In this work, caching optimization is formulated as a sequential decision-making problem and addressed using a DQN agent. The learning rate λ determines the magnitude of policy updates based on newly observed transitions. A value of 0.05 is selected to ensure stable convergence while preserving sufficient adaptability to dynamic content request patterns. The discount factor γ is set to 0.1 to prioritize immediate cache hits, which is particularly important in latency-sensitive vehicular environments where delayed rewards have limited practical value.

An ϵ-greedy exploration strategy is adopted to balance exploration and exploitation during training. The exploration rate is initially set to ϵ=1.0 to encourage exhaustive exploration of the action space and is gradually decayed by a factor of 0.995 until reaching a minimum value of 0.01. This mechanism ensures continued but limited exploration, preventing premature convergence to suboptimal caching policies.

In this study, an episode corresponds to one complete interaction cycle between the DQN-based caching agent and the IoV environment under a fixed mobility realization. Each episode consists of multiple decision epochs, during which the agent observes the current system state, selects a caching action, receives a reward, and transitions to a new state. Consequently, although the total number of episodes is limited, the DQN agent is trained over several thousand state-action transitions in aggregate, ensuring sufficient learning depth beyond short toy scenarios.

The DQN model is selected due to its suitability for discrete cache placement actions and its relatively low computational overhead. Compared to policy-gradient methods such as Proximal Policy Optimization (PPO) or Trust Region Policy Optimization (TRPO), DQN offers a simpler training pipeline and lower inference complexity, making it more practical for edge-assisted IoV deployments with constrained computational resources. While more advanced DRL algorithms may further improve performance in large-scale or continuous-action settings, their investigation is left for future work (see Table 2).

#### 4.2.3. Network Parameters

Network parameters define the communication characteristics between entities in the simulated IoV environment. A nominal channel bandwidth of 10 MHz is adopted to represent a typical allocation for vehicular wireless communications (e.g., DSRC or cellular V2X). In this study, bandwidth is used as an abstract parameter to differentiate link capacities rather than to explicitly model physical-layer throughput, which is outside the scope of caching optimization.

To account for variable transmission latency caused by congestion, channel contention, and signal impairments, a delay factor of 2.5 is introduced as a multiplicative coefficient applied to baseline link delays. This factor enables relative delay differentiation across links without relying on a detailed protocol-level latency model, allowing the simulation to focus on comparative caching performance.

Signal-to-Interference-plus-Noise Ratio (SINR) values are configured to reflect heterogeneous link qualities within the network. A value of 20 dB is assigned to V2I links to represent stable infrastructure-supported communication, while V2V links operate at 10 dB to capture average peer-to-peer conditions. Inter-cluster links are assigned a lower SINR of 5 dB to emulate weaker and less reliable connectivity across cluster boundaries.

A fixed delay of 5 s is applied to remote server content retrieval to emulate high-latency access to cloud or backbone networks. This value is not intended to model exact TCP/IP timeout behavior but rather to represent a significantly larger delay compared to edge-level retrieval, thereby highlighting the benefit of proactive caching and local content availability (see Table 3).

#### 4.2.4. Model Parameters

This subsection describes the configuration of the proposed hybrid LSTM–Transformer model used for content request prediction. The selected parameters are designed to balance prediction accuracy and training efficiency while accounting for the computational constraints typical of edge-assisted IoV environments. The hyperparameters in Table 4 were selected to balance predictive performance and computational overhead suitable for edge-assisted IoV deployment. We used a 20% validation split to monitor generalization during training and applied dropout to mitigate overfitting in the compact setting. For fair comparison, the Transformer-only baseline uses the same Transformer configuration and training protocol as the Transformer component of the hybrid model.

An input sequence length of T=10 is adopted to capture short-term temporal dependencies in recent content request history without introducing excessive input dimensionality. This window size provides sufficient temporal context for prediction while maintaining stable and efficient training. The LSTM layer is configured with 50 hidden units to model sequential patterns, followed by a Transformer encoder with 3 attention heads and a feed-forward dimension of 128 to capture non-local dependencies and contextual relationships across time steps. The Transformer component consists of a single encoder layer, which is sufficient to capture global dependencies in the considered input sequences while maintaining low computational overhead.

A dropout rate of 0.1 is applied to mitigate overfitting, particularly given the compact size of the training dataset. The prediction model is trained using the Adam optimizer [32], a standard adaptive gradient-based optimization algorithm widely used for training deep neural networks. Mean Squared Error (MSE) is used as the loss function to penalize deviations between predicted and actual content request intensities.

The model is trained for 20 epochs with a batch size of 32. This number of epochs is sufficient to ensure convergence of the prediction model while avoiding overfitting, as verified through validation monitoring. A validation split of 20% is used to track generalization performance during training. For fair comparison, the Transformer-only baseline adopts the same input sequence length, attention head count, feed-forward dimension, optimizer, and training configuration as the Transformer component of the proposed hybrid model.

### 4.3. Evaluation Metrics

To evaluate the effectiveness of the proposed hybrid model, both system-level caching performance and the accuracy of content request prediction are assessed. A well-defined evaluation framework is adopted to capture improvements in content delivery efficiency, as well as the predictive capability of the learning model under controlled simulation conditions.

The evaluation relies on two complementary categories of performance indicators. System-level metrics, such as cache hit rate and content acquisition delay, reflect the operational efficiency of the caching strategy. In parallel, classification metrics—including accuracy, precision, recall, and F1-score—are used to assess the effectiveness of the model in identifying popular content items that should be prioritized for caching.

#### 4.3.1. Performance Metrics

The performance of caching mechanisms in vehicular networks directly impacts system efficiency and user experience. Effective caching strategies reduce access latency, alleviate network congestion, and improve delivery reliability. Accordingly, system performance is quantified using two primary indicators: cache hit rate and average content acquisition delay.

The cache hit rate is defined as the ratio of the number of content requests satisfied by local caches (cluster vehicles or RSUs) to the total number of content requests:

(9) $$\mathrm{Cache}\;\mathrm{Hit}\;\mathrm{Rate}=\frac{\mathrm{Number}\;\mathrm{of}\;\mathrm{Hits}}{\mathrm{Total}\;\mathrm{Number}\;\mathrm{of}\;\mathrm{Requests}}.$$

A higher cache hit rate indicates that a larger fraction of requests is served locally, thereby reducing backhaul usage and improving perceived service responsiveness. It should be noted that this metric reflects caching efficiency under the given experimental configuration and does not imply global optimality in large-scale deployments.

In addition to cache hit rate, the average content acquisition delay measures system responsiveness. This metric captures the time required to retrieve requested content from different sources, including intra-cluster caches, RSUs, neighboring clusters, or remote servers, under heterogeneous network conditions. Lower acquisition delay values indicate faster content delivery and improved quality of service. Together, these two metrics provide a comprehensive view of caching effectiveness and support comparative analysis among different strategies.

#### 4.3.2. Classification Metrics

Accurate prediction of content popularity is essential for enabling proactive caching decisions. In this work, content popularity prediction is treated as a binary classification problem, where content items are labeled as either popular or less popular based on predicted request intensity. Standard classification metrics are employed to quantify prediction performance.

Let TP, TN, FP, and FN denote true positives, true negatives, false positives, and false negatives, respectively. The corresponding evaluation metrics are defined as follows:

(10) $$\mathrm{Accuracy}=\frac{\mathrm{TP}+\mathrm{TN}}{\mathrm{TP}+\mathrm{TN}+\mathrm{FP}+\mathrm{FN}}.$$

(11) $$\mathrm{Precision}=\frac{\mathrm{TP}}{\mathrm{TP}+\mathrm{FP}}.$$

(12) $$\mathrm{Recall}=\frac{\mathrm{TP}}{\mathrm{TP}+\mathrm{FN}}.$$

(13) $$F1\text{-}\mathrm{Measure}=\frac{2\;\mathrm{Precision}\;\mathrm{Recall}}{\mathrm{Precision}+\mathrm{Recall}}.$$

These metrics evaluate the model’s ability to correctly identify high-demand content while avoiding unnecessary cache allocation. Statistical significance and convergence behavior of the observed performance gains are analyzed in the subsequent results section.

### 4.4. Results and Performance Analysis

In this subsection, we analyze the results obtained from the simulation-based evaluation of the proposed hybrid content request prediction and caching framework. The analysis focuses on both the learning behavior of the reinforcement learning component and the resulting system-level caching performance. The dataset used for content request prediction is generated from request traces inspired by the Telecom Italia Big Data Challenge dataset [33], and includes request data for ten content items under controlled conditions.

#### 4.4.1. Convergence Behavior of the DQN-Based Caching Agent

The effectiveness and stability of the proposed DQN-based caching strategy are first evaluated through convergence analysis. Two key metrics are monitored during training: the DQN training loss and the episode return, which together reflect the learning dynamics and policy improvement of the agent.

**Figure 5:**
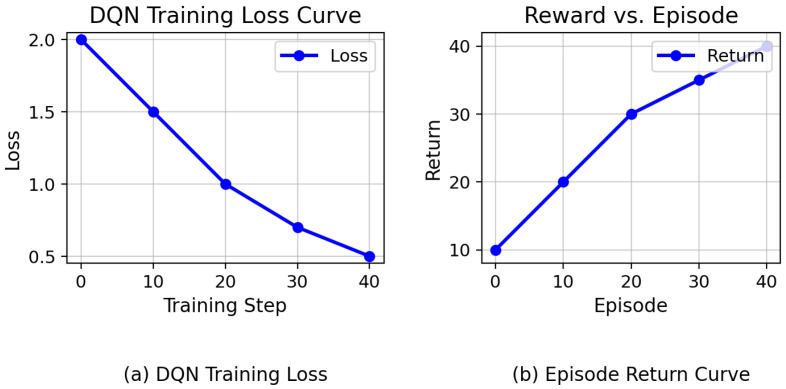
(**a**) DQN loss vs. training steps, (**b**) Episode return vs. episode index.

As illustrated in Figure 5a, the training loss decreases monotonically as training progresses, indicating that the DQN successfully minimizes the temporal-difference (TD) error and converges toward a stable approximation of the action-value function. This behavior confirms that the experience replay mechanism and target network updates contribute to stable learning and prevent divergence.

Figure 5b shows the evolution of the episode return across training episodes. The steadily increasing trend demonstrates that the agent progressively learns a caching policy that maximizes the composite reward, which jointly accounts for cache hit rate, latency reduction, and resource savings. After an initial exploration phase, the return curve stabilizes, indicating convergence toward a consistent policy.

Overall, the convergence patterns observed in Figure 5 validate the robustness of the proposed reinforcement learning framework. Despite the dynamic vehicular environment and the multi-objective reward formulation, the agent exhibits stable learning behavior across training, providing a reliable foundation for subsequent performance evaluation.

#### 4.4.2. System-Level Performance Results

The system-level performance of the proposed hybrid model is evaluated using cache hit ratio and average content acquisition delay. During the testing phase (episodes 500–1000), the proposed approach achieves a high cache hit ratio, indicating that the majority of content requests are served locally by cluster vehicles or RSUs without reliance on remote servers. The average content acquisition delay is correspondingly reduced, reflecting improved responsiveness of the caching system.

**Figure 6:**
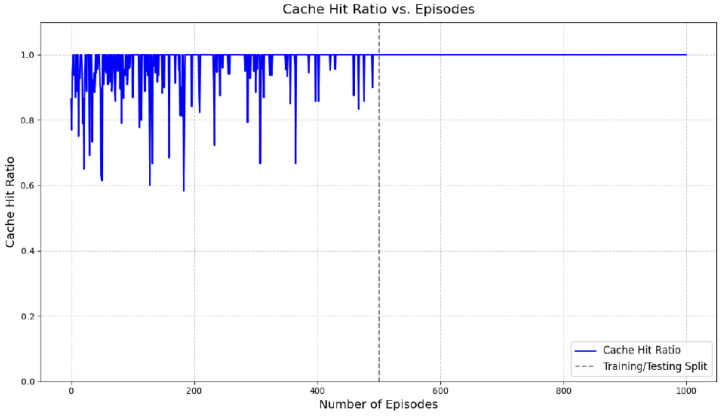
Variation of cache hit ratio across training and testing episodes.

**Figure 7:**
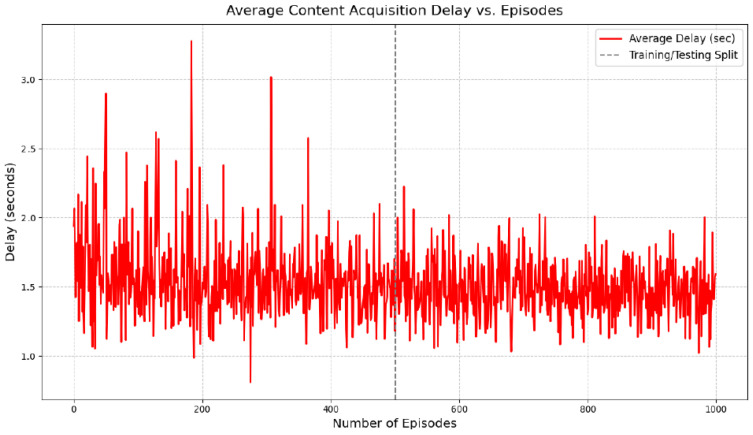
Variation of average content acquisition delay across training and testing episodes.

As shown in Figure 6, the cache hit ratio exhibits fluctuations during the training phase (episodes 0–500), which is expected due to the exploration–exploitation trade-off inherent in reinforcement learning. After convergence, the hit ratio stabilizes at a high level during the testing phase. Similarly, Figure 7 shows that the average content acquisition delay decreases over time and becomes more stable once training is completed.

It is important to note that the observed near-perfect cache hit ratio reflects optimal convergence under the constrained experimental setting and should be interpreted as a comparative performance indicator rather than an absolute guarantee in large-scale deployments. Nevertheless, the results demonstrate the effectiveness of integrating accurate content request prediction with adaptive reinforcement learning-based caching decisions in dynamic IoV environments.

### 4.5. Confusion Matrix Analysis

**Figure 8:**
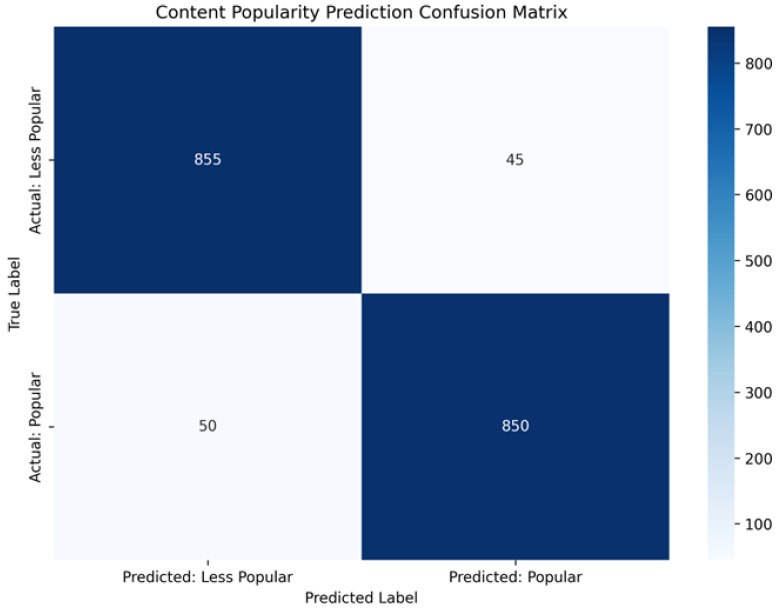
Confusion Matrix for Content Popularity Prediction (aggregated over the testing phase, episodes 500–1000, across decision epochs and content evaluations).

**Figure 9:**
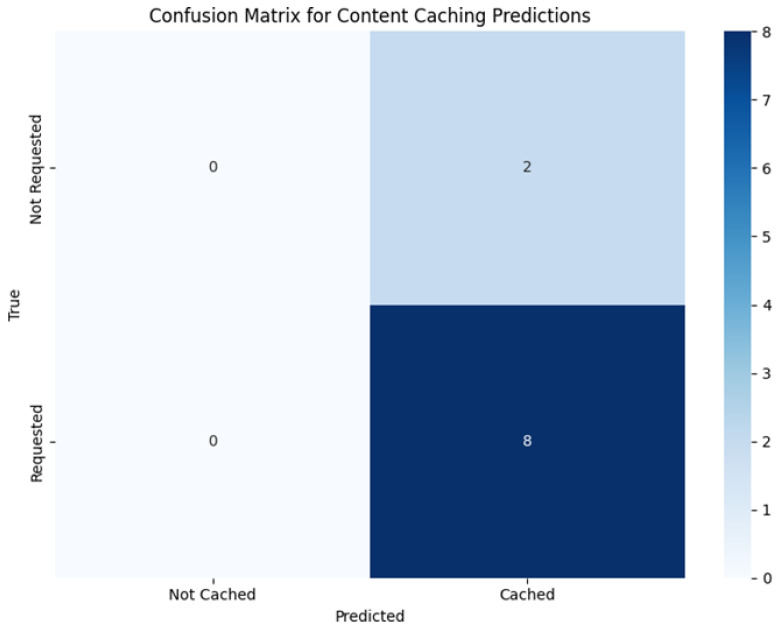
Catalog-Level Confusion Matrix for Caching Decisions in the proof-of-concept scenario (K=10 content items; cached vs. not cached, computed at a given decision epoch).

To further examine the predictive behavior of the proposed hybrid model, confusion matrix analysis is conducted based on the testing phase (episodes 500–1000). Figure 8 and Figure 9 illustrate the confusion matrices for content popularity prediction and caching decision prediction, respectively. This analysis complements the quantitative evaluation metrics presented earlier by providing insight into the distribution of correct and incorrect classification outcomes under controlled simulation conditions.

The two confusion matrices in Figure 8 and Figure 9 are computed at different granularities. Figure 8 (popularity prediction) aggregates binary classification outcomes across the full testing phase (episodes 500–1000) over multiple decision epochs and content evaluations, which explains the larger total counts. In contrast, Figure 9 (caching decision) is computed at the catalog level over the K=10 content items (cached vs. not cached) under the corresponding caching decision snapshot; hence, the total count is 10. Request-level performance is evaluated separately using hit ratio and content acquisition delay, which aggregate over all generated request events during testing.

The predictor outputs continuous per-content scores ${\hat{p}}_{t+1}$ for the next decision epoch. To compute confusion matrices, we convert these scores into binary labels using a consistent top-κ rule. At each epoch *t*, we derive a ground-truth demand vector $p_{t+1}$ from the realized requests in the next decision epoch/window (e.g., empirical request counts or normalized frequencies), and label content *k* as popular if it belongs to the top-κ entries of $p_{t+1}$; otherwise it is labeled less popular. The same top-κ rule is applied to ${\hat{p}}_{t+1}$ to obtain predicted binary labels. Figure 8 aggregates these (true,predicted) labels across all testing epochs and contents. For Figure 9, the caching decision is evaluated at a given decision epoch at the catalog level by labeling each content item as cached vs. not cached based on whether it is stored in any node (vehicles/RSU), and comparing this decision against the corresponding occurrence label in the same epoch (requested vs. not requested, as shown in Figure 9). We set κ=3 (a small popular set for K=10 under constrained cache capacity) for all methods to ensure consistent evaluation; ties at the cutoff are broken by descending score, then content index.

Figure 8 presents the confusion matrix for content popularity prediction. The results indicate that the model is able to distinguish between popular and less popular content with a high degree of consistency, achieving an overall accuracy of 94.7%. The relatively high numbers of true positives (850) and true negatives (855) suggest that the majority of content items are correctly classified. This behavior is important for proactive caching, as it enables frequently requested content to be prioritized while avoiding unnecessary allocation of cache resources to low-demand items. The observed precision of 95.0% implies a limited number of false positives, whereas the recall of 94.4% indicates that most high-demand content items are successfully identified. Together, these results suggest a balanced prediction behavior without pronounced bias toward either class.

Figure 9 illustrates the confusion matrix for caching decision prediction, which evaluates how effectively the predicted popularity information is translated into cache placement decisions. The absence of false negatives (FN = 0) indicates that all content items requiring caching are correctly identified in this experimental setting, resulting in a recall of 100%. While this outcome reflects conservative caching behavior, it is consistent with the objective of avoiding missed caching opportunities in latency-sensitive vehicular environments. At the same time, the presence of two false positives leads to a precision of 80%, indicating that some content items may be cached despite lower demand.

Although the resulting accuracy (80%) and F1-score (88.9%) are obtained under a limited-scale dataset, they demonstrate that the model maintains a reasonable balance between over-caching and under-caching. Importantly, these confusion matrix results should be interpreted as supportive evidence of the model’s decision behavior rather than as a standalone indicator of overall system superiority. The implications of these predictive outcomes on cache efficiency and retrieval delay are further examined through direct performance comparisons with baseline and state-of-the-art approaches in the following subsection.

### 4.6. Comparison with State-of-the-Art Approaches

To evaluate the relative effectiveness of the proposed hybrid caching framework, its performance is compared with four representative baseline strategies: an LSTM-only prediction-based caching approach (CCCRP) [16], a Transformer-only model, and two classical heuristic policies, namely Least Frequently Used (LFU) and Least Recently Used (LRU). These baselines are selected to cover both learning-based and non-learning-based caching paradigms commonly adopted in IoV systems. The comparison focuses on key performance indicators, including cache hit ratio, average content acquisition delay, and adaptability to variations in cache capacity and content popularity distributions.

**Figure 10:**
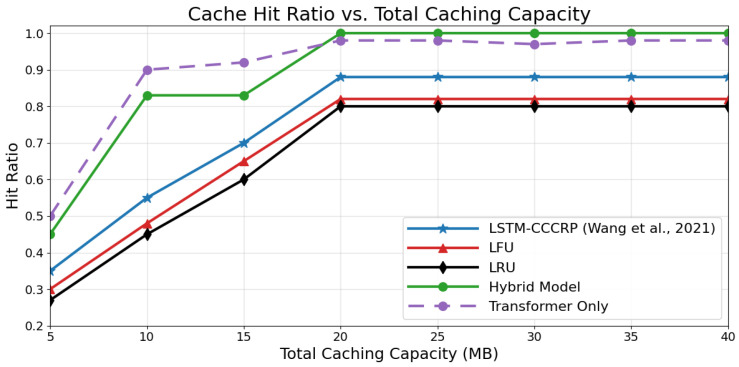
Cache hit ratio versus total caching capacity for LSTM-CCCRP [16], LFU, LRU, Transformer Only, and the Hybrid Model.

Figure 10 illustrates the impact of total caching capacity on cache hit ratio for all evaluated strategies. As cache capacity increases, the proposed hybrid LSTM–Transformer model achieves consistently high hit ratios and reaches saturation at moderate cache sizes. This behavior indicates that the hybrid model can effectively exploit available cache resources through informed, data-driven caching decisions. At lower cache capacities (5–10 MB), the Transformer-only model exhibits competitive performance, likely due to its ability to capture short-term contextual patterns. However, as cache capacity increases, its performance plateaus below that of the hybrid model, suggesting limitations in modeling longer-term temporal dependencies. In contrast, LFU and LRU exhibit limited scalability, with hit ratios saturating below 0.85, reflecting their inability to adapt to evolving request patterns. The LSTM-only model provides moderate improvements over heuristic approaches but remains consistently below the hybrid framework.

**Figure 11:**
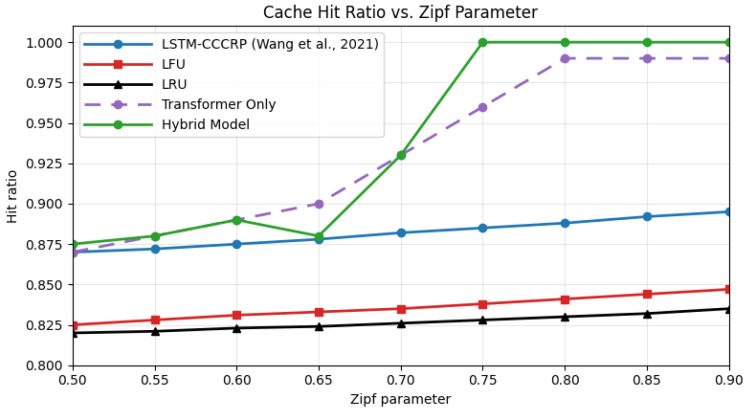
Cache hit ratio versus Zipf parameter for LSTM–CCCRP [16], LFU, LRU, Transformer Only, and the Hybrid Model.

To further examine adaptability under different demand distributions, Figure 11 reports cache hit ratios across varying Zipf parameters. As content popularity becomes more skewed, all learning-based approaches benefit from increased predictability. The hybrid model maintains stable and high performance across all tested Zipf values, indicating robust behavior under non-uniform access patterns. The Transformer-only model performs competitively at higher skew levels but remains slightly below the hybrid approach. In contrast, LFU and LRU show limited sensitivity to changes in the Zipf parameter, reinforcing their lack of adaptability in dynamic environments.

**Figure 12:**
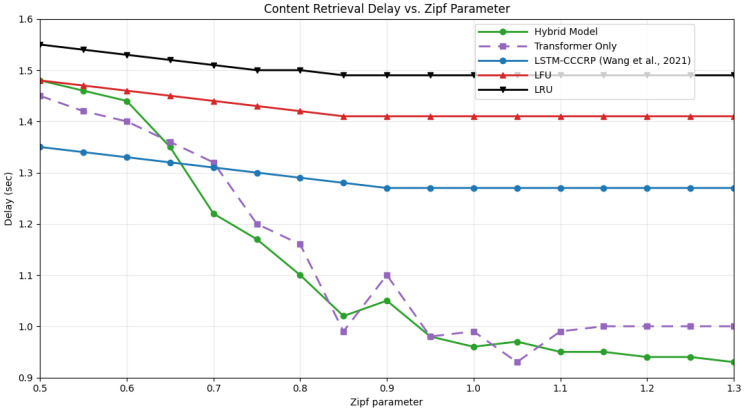
Average content acquisition delay versus Zipf parameter for Hybrid, Transformer Only, LSTM–CCCRP [16], LFU, and LRU caching strategies.

Figure 12 compares the average content acquisition delay across different Zipf parameters. As content demand becomes increasingly concentrated, the hybrid model demonstrates a consistent reduction in retrieval delay, reflecting its ability to prioritize frequently requested content and optimize cache placement. While the Transformer-only and LSTM-only models also benefit from increased skew, their delay remains higher than that of the hybrid framework. Heuristic strategies exhibit minimal improvement, with delays remaining comparatively high due to their reactive nature. These observations suggest that predictive, learning-based caching can offer latency benefits under controlled IoV scenarios.

**Figure 13:**
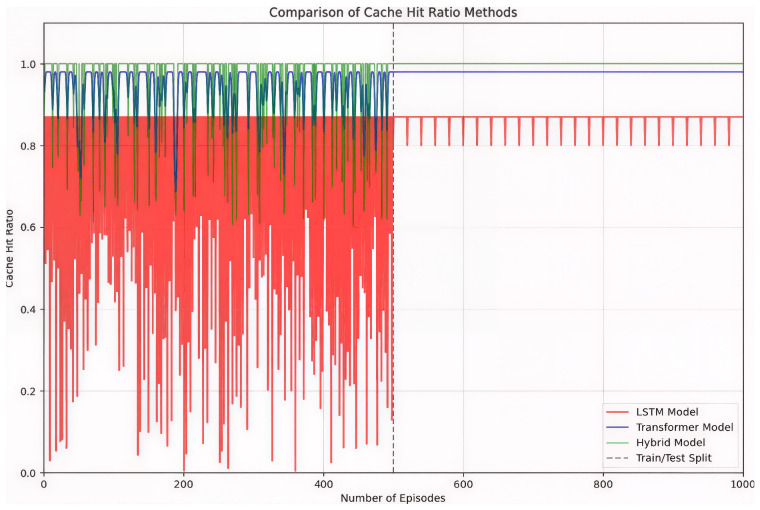
Cache Hit Ratio Across Episodes for LSTM, Transformer, and the Hybrid Model.

The temporal stability of learning-based caching strategies is further examined in Figure 13, which presents cache hit ratios across training and testing episodes for the LSTM-only, Transformer-only, and hybrid models. The hybrid approach maintains a stable performance profile throughout the learning process, while the Transformer model converges smoothly but at a slightly lower level. The LSTM-only model exhibits higher variance during training and reduced generalization during testing, highlighting the benefit of integrating both sequential memory and attention mechanisms.

Table 5 summarizes the comparative performance of all evaluated approaches. Classical heuristics (LRU/LFU) achieve lower hit ratios and higher delays, reflecting their limited adaptability. The LSTM-only model improves prediction accuracy but offers modest delay reduction. The Transformer-only model further enhances predictive accuracy and responsiveness. The proposed hybrid model achieves a balanced performance across all metrics under the given experimental configuration, combining high cache hit ratio, competitive delay, and improved prediction accuracy at moderate cache sizes. These results indicate that integrating temporal modeling with attention mechanisms can enhance caching decisions in dynamic vehicular environments. However, the observed performance gains should be interpreted within the scope of the simulated setting and do not imply guaranteed optimality under large-scale real-world deployments.

### 4.7. Discussion and Practical Implications

The obtained results provide useful insights into the end-to-end behavior of the proposed hybrid architecture under controlled simulation conditions. The evaluation, conducted with a 15-vehicle setup, demonstrates that the model can achieve high cache hit ratio, strong prediction accuracy, and low content acquisition delay, highlighting its suitability for real-time caching decision support in IoV environments. From a computational perspective, the hybrid design—combining an LSTM layer with 50 hidden units and a Transformer encoder with three attention heads—maintains moderate model complexity, which is compatible with execution on edge servers or RSUs equipped with sufficient processing resources. However, the current study does not explicitly evaluate large-scale vehicular densities, and performance may be affected by practical constraints such as limited wireless bandwidth, finite cache capacity, and increased request concurrency. To address these challenges in more demanding scenarios, extensions such as batch inference, adaptive cache management, or multi-tier prediction and caching across edge and cloud layers could be considered. Consequently, while the proposed approach shows promising behavior within the evaluated setting, further validation using larger datasets, denser traffic scenarios, and real-world mobility traces is required to comprehensively assess its scalability and robustness.

While the proposed framework integrates a hybrid LSTM–Transformer predictor with a DQN-based caching controller, the present evaluation is intended as an end-to-end proof-of-concept under a controlled simulation setting. To isolate the incremental contribution of prediction-in-the-loop control beyond reinforcement-learning-based caching alone, an extended evaluation should include a full RL ablation set under identical training conditions, such as a DQN baseline without prediction input, as well as DQN variants coupled with single predictors (LSTM-only and Transformer-only). Accordingly, the improvements reported in this paper should be interpreted as end-to-end gains of the integrated framework relative to classical caching heuristics and predictor baselines, rather than as a component-wise attribution of gains. In addition, while we motivate the serial LSTM-first, Transformer-later design as an inductive-bias choice for nonstationary request sequences, an explicit architecture-order ablation (e.g., Transformer-first and parallel fusion variants under matched training budgets) remains an important direction for extended evaluation.

Many IoV services generate recurring yet highly time- and location-dependent data demands, where missed opportunities during short vehicle–RSU contact times can translate into late deliveries and unnecessary remote fetches. Representative examples include cooperative driving and cooperative perception services (e.g., exchanging perception information among vehicles and infrastructure), distribution of high-definition map tiles and frequent incremental updates for automated driving, roadside dissemination of localized traffic/event information (e.g., incident alerts, work-zone warnings, and signal phase and timing), and infotainment delivery in transient hotspots. These application classes are widely recognized as latency-sensitive and vulnerable to imperfect V2X conditions such as packet loss, interference, and dynamic topology changes, which motivates proactive edge-side preparation rather than purely reactive caching [34,35,36,37]. In this context, the proposed framework is relevant because it couples demand forecasting with cache control in a single loop: forecasting provides an anticipatory signal about which items are likely to be requested soon, while the RL-based controller adapts placement/replacement decisions to current cache occupancy and operational constraints. This design intent aligns with recent IoV research trends that combine edge intelligence and learning-based control to support time-critical services under mobility and resource constraints, including DRL-based edge-end collaboration for service execution and integrated learning/caching in dynamic IoV deployments [38,39].

## 5. Conclusions

This paper presented a hybrid deep learning framework that integrates Long Short-Term Memory (LSTM) networks with Transformer encoders for predictive content caching in Internet of Vehicles (IoV) environments. By jointly modeling short-term temporal dynamics and longer-range dependencies in content request patterns, the proposed architecture enables effective forecasting of content popularity. These predictions are subsequently exploited by a DQN-based caching policy to support proactive and adaptive cache placement at both vehicles and roadside units.

Simulation results obtained under controlled experimental conditions indicate that the proposed hybrid approach can improve cache hit ratio, reduce content acquisition delay, and enhance prediction accuracy when compared with representative baseline strategies, including LSTM-only, Transformer-only, LFU, and LRU. The observed performance gains support the effectiveness of the proposed end-to-end framework under the evaluated setting, in which predicted popularity information is used to inform learning-based cache control. A more granular attribution of gains to individual components of the prediction-in-the-loop design (e.g., RL-only and single-predictor-in-the-loop variants) is an important direction for extended evaluation.

The primary contribution of this work lies in the end-to-end integration of deep content request prediction with reinforcement learning-based caching decisions, providing a coherent and flexible architecture for context-aware content delivery in dynamic vehicular networks. Nevertheless, the current evaluation is limited to a compact proof-of-concept simulation environment with synthetic request generation, and the reported results should be interpreted as comparative indicators under identical conditions rather than as guarantees for large-scale, high-density IoV deployments. Future work will therefore extend the validation by scaling the simulator by one to two orders of magnitude (e.g., from 15 vehicles to hundreds to thousands of vehicles and from 10 content items to hundreds to thousands) and evaluating multi-RSU deployments using trace-driven mobility and request generation. In addition, future work will investigate more advanced learning paradigms such as federated learning, cooperative multi-agent caching, and cross-layer optimization mechanisms. Overall, this study contributes insights into the design of learning-assisted caching strategies for IoV systems and lays the groundwork for further research toward scalable and practical deployments.

## Figures and Tables

**Table 1:** System parameters used in the simulation setup.

Parameter	Value
Number of vehicles	15
Number of clusters	2
Number of caching nodes	3
Cache capacity per vehicle	5 MB
RSU cache capacity	10 MB
Number of unique content items	10
Zipf parameter	0.7
Total simulation episodes	1000

**Table 2:** Reinforcement learning hyperparameters used for training.

Parameter	Value
Learning rate (λ)	0.05
Discount factor (γ)	0.1
Initial exploration rate (ϵ)	1.0
Exploration decay rate	0.995
Minimum exploration value	0.01

**Table 3:** Network communication parameters used in the simulation.

Parameter	Value
Bandwidth	10 MHz
Delay factor	2.5
V2V SINR	10
V2I SINR	20
Inter-cluster SINR	5
Remote server delay	5.0 s

**Table 4:** Model parameters used in the proposed prediction model.

Parameter	Value
Input sequence length	10
LSTM units	50
Attention heads	3
Feed-forward dimension	128
Dropout rate	0.1
Loss function	MSE
Optimizer	Adam
Training epochs	20
Batch size	32
Validation split	0.2

**Table 5:** Comparison of caching performance metrics for baseline (LRU/LFU) and learning-based models. Values with “∼” indicate approximate ranges. Accuracy is N/A for LRU/LFU since these baselines do not output predictions. “Min. Cache” denotes the minimum cache capacity needed to reach the listed hit rate and delay.

Model	Hit Rate	Delay (s)	Accuracy	Min. Cache
LRU/LFU	∼0.75–0.85	1.4–1.5	N/A	>30 MB
LSTM Model	∼0.85–0.90	∼1.4	∼85–90%	∼25 MB
Transformer Model	0.98	∼1.2	91.2%	∼20 MB
Hybrid Model	1.0	∼1.3	∼95%	∼20 MB

## Data Availability

The dataset analyzed in this study is publicly available.

## Abbreviations

The following abbreviations are used in this manuscript:

IoV	Internet of Vehicles
ITS	Intelligent Transportation Systems
V2I	Vehicle-to-Infrastructure
V2V	Vehicle-to-Vehicle
V2X	Vehicle-to-Everything
RSU	Roadside Unit
RL	Reinforcement Learning
DRL	Deep Reinforcement Learning
TD	Temporal-Difference
DQN	Deep Q-Networks
LSTM	Long Short-Term Memory
RNN	Recurrent Neural Network
LFU	Least Frequently Used
LRU	Least Recently Used
CV	Cluster Vehicles
